# Two-Source Capture-Recapture Method to Estimate the Incidence of Acute Flaccid Paralysis in the Marches Region (Italy)

**DOI:** 10.3390/ijerph17249400

**Published:** 2020-12-15

**Authors:** Pamela Barbadoro, Aurora Luciani, Matteo Ciotti, Marcello Mario D’Errico, on behalf of the AFP Working Collaborative Group

**Affiliations:** 1 Department of Biomedical Science and Public Health, Università Politecnica delle Marche, via Tronto 10/A, 6, 60121 Ancona, Italy; igiene.marche@gmail.com (A.L.); musa@univpm.it (M.C.); igiene.ricerca@gmail.com (M.M.D.); 2 Infection Control Unit, AOU Ospedali Riuniti di Ancona, via Conca, 60126 Torrette di Ancona, Italy; 3 School of Hygiene and Preventive Medicine, Department of Biomedical Science and Public Health, Università Politecnica delle Marche, via Tronto 10/A, 6, 60121 Ancona, Italy

**Keywords:** acute flaccid paralysis, poliomyelitis, surveillance systems, sensitivity, epidemiologic surveillance/methods, epidemiologic methods, socioeconomic factors, Italy

## Abstract

A combination of high infant immunization coverage and surveillance of acute flaccid paralysis (AFP) cases, plays a critical role in polio eradication. This study aimed to estimate the incidence of AFP, to evaluate the completeness of AFP ascertainment during the years, age groups and gender, and to define the main associated diagnosis among children aged under 15 in the Marches region of Italy. Analysis was performed on data from the active AFP surveillance system and the hospital discharge records in the 2006–2014 period. The two-source capture-recapture method was applied. After cross-validation, 30 AFP compatible conditions as defined by the WHO were identified, with an incidence estimate of 1.91 cases of AFP per 100,000 children under 15 years (95% CI = 1.4–2.6/100,000). Guillain–Barrè syndrome was the most common diagnosis. A significant difference (*p* < 0.05) has been registered in the estimated probability of case ascertainment in females. The reasons for the lower reporting of cases in females are unknown. Specific research and the implementation of a more sensitive surveillance system are essential in verifying potential inequalities and to succeed in the polio eradication initiative.

## 1. Introduction

In May 1988, the World Health Assembly launched the Global Polio Eradication Initiative (GPEI) aimed to achieve the goal of global eradication of poliomyelitis by 2000. The goal was defined as no cases of clinical poliomyelitis associated with wild poliovirus and no wild poliovirus found worldwide. The strategy subsequently adopted by the World Health Organization (WHO) relies on four key strategies: high infant immunization coverage, mass campaigns, active surveillance for wild poliovirus and target “mop up” campaigns [1,2].

Europe has been declared polio free since 2002. In Italy the last case of poliomyelitis due to transmission of indigenous wild poliovirus occurred in 1982 [3]. In 2018 polio was still declared a Public Health Emergency of International Concern (PHEIC) by the WHO due to Poliovirus (PV) reintroduction through populations movements, such as cross border spreading in both direction from Pakistan to Afghanistan [4]. However, the WHO has highlighted that it has been four years since there was any international spread of wild types of poliovirus (WPV) outside of these two epidemiologically linked countries [5,6]. Since then, in order to detect any virus importation, a reinforcement of PV surveillance was encouraged everywhere to prevent the risk that international spread may go undetected [5,6,7]. Therefore, in polio-free countries it is important to guarantee high quality surveillance until polio is eradicated globally, to prevent the risk of importation of cases from areas where the disease is still endemic [8,9,10],

Active surveillance for acute flaccid paralysis (AFP) is considered a very sensitive system to rapidly detect, report and investigate confirmed poliomyelitis cases. In countries in polio-free regions, AFP surveillance should be evaluated through indicators, such as by sensitivity of surveillance and completeness of case investigation [11]. Indeed, the number of cases reported every year is used as an indicator of a country’s ability to detect polio, even in polio-free countries. 

The incidence in any population of AFP cases due to conditions other than polio (the non-polio AFP rate) is estimated to be one case per 100,000 children aged under 15 years [11]. A country’s surveillance system should be sensitive enough to detect at least 1 case of AFP for every 100,000 children aged under 15, even in the absence of polio. Also, a surveillance system should ensure the completeness of case investigation. GPEI suggests that all AFP cases should have a full investigation (virologic and clinical) with at least 80% of AFP cases having “adequate” stool specimens collected [11]. In 2017, the Italian Ministry of Health declared that AFP surveillance had shown different critical aspects, including the under-notification of cases (estimated to be from 20% to 60%), estimated through the comparison between the notified data from the national AFP surveillance and the data the hospital discharge records (HDRs).

The aims of this study were to estimate the incidence of AFP, to evaluate the completeness of AFP ascertainment by year, age groups and gender, and to define the main associated diagnosis among children aged under 15 in the Marches Region of Italy.

## 2. Methods

### 2.1. Organization of AFP Surveillance in Italy

In Italy, the surveillance of AFP was extended to a national level in 1997 and carried out by the National Institute of Health (ISS) and the Ministry of Health (MOH) through 20 Regional Reference Centres (RRCs). An AFP case is defined as a child aged under 15 showing clinical acute onset of flaccid paralysis in one or more limbs, or acute onset of bulbar paralysis [12].

Clinical samples (consisting of two stool specimens and one sample of serum) are sought for each case and sent to the ISS for virologic investigations. After case notification, a follow-up questionnaire is required to be sent to the ISS and MOH to determine whether residual paralysis or death occurred 60–90 days after diagnosis, as well as to clarify the conclusive diagnoses. Virologic investigations are usually performed by ISS [13].

### 2.2. Organization of AFP Surveillance at the Regional Level

The Chair of Hygiene at the Polytechnic University of the Marche is the RRC for the same region. The Marches are one of the twenty regions of Italy. The name of the region derives from the plural name of marca, originally referring to the medieval March of Ancona and to the nearby marches of Camerino and Fermo, representing its cross-border nature. The region is located in the Central area of the country on the Adriatic Sea, and has about 1.5 million inhabitants. On a two-weeks basis designated staff contacts by phone the reference doctors of 25 Operative Units (15 of pediatrics, 3 of infectious diseases, 7 of neurology/child neuropsychiatry) belonging to the network. For every AFP case the management is activated according to the directives of the ISS and MOH. 

### 2.3. Data Sources and Diseases under Study

The study was carried out retrospectively with data from 2006 to 2014. The primary data source was the regional database of AFP cases originating from notifications by phone to the RRC of Marche. The secondary data source comprised hospital discharge records (HDRs) including a diagnosis consistent with AFP-compatible conditions as defined by the WHO. Characteristics of paralytic polio are fever at onset, asymmetric flaccid paralysis, rapid progression of paralysis, residual paralysis after 60 days and preservation of sensory nerve function. The most common differential diagnoses of AFP include Guillain-Barré syndrome, paralytic poliomyelitis, and transverse myelitis [14]. Therefore, the study focused on 29 diagnostic ICD-9 codes (main or secondary) listed in Appendix A. We analyzed only the AFP cases occurring in children aged <15 years and the HDRs belonging to ordinary hospitalization in the same period by the same hospitals involved in surveillance. 

The authorization from the local Ethics Committee was not required, as the program belongs to a national ministerial project considering the WHO directives for the achievement of the poliomyelitis eradication (WHA 41.28 of May 1988). Research carried out on humans was performed in compliance with the Helsinki Declaration.

The two-source capture-recapture method was used to assess the incidence of AFP cases, the sensitivity of the surveillance system to detect all AFP cases and the completeness of case investigation [15,16,17,18]. Hospital records were linked to the regional database of notifications. The likelihood of association between two records was based on a core set of identifiers (age, sex of the patient, and month and year of notification or hospital admission). Linked cases were allocated to the year of the first known date of notification or hospital admission (in case of absence of a notification) and laboratory culture confirmation date (for notified cases). Estimates were stratified by year of diagnosis, age group (0–3 years, 4–7 years, 8–11 years, and 12–15 years), ICD9-CM diagnosis code, and gender. The sensitivity of the system was defined as the number of notified cases divided by the total number of cases as estimated by the capture-recapture method.

In this study we compared cases from two sources to estimate the total number of cases, based on Formula (1):

*N* = ab/c (1)

where *N* is the total number of cases, a is the total number of cases determined from the primary source (regional database of AFP cases notified in Marches), b is the total number of cases ascertained from the secondary source (hospital discharge records) and c is the number of cases established by both sources. In the present study, the number of AFP estimated cases has been calculated using the Chapman modification to the Lincoln–Petersen estimator for the capture-recapture method, as follows (2):

*N* = [(a + 1)(b + 1)/(c + 1)] − 1 (2)

Estimated completeness was calculated based on Formula (3):

d = a/*N* × 100 (3)

where d is the estimated completeness, a is the total number of cases determined from the primary source (regional database of AFP cases notified in Marches), and *N* is the total number of estimated cases. The difference between ascertained/estimated number of events has been assessed by the chi-squared test. The 95% CI for the population size was calculated using the ciChapman function from the recapr package, with the default bootstrap method. Analyses were performed with the r software. [19] The level of significance was set at 0.05.

## 3. Results

The regional database of AFP cases notified to the RRC included information about six AFP cases in the 2010–2014 period, equivalent to an annual incidence of 0.54 per 100,000 children aged under 15 years (95% CI = 0.25–1.86/100,000). According to the recommended WHO target level, a minimum of 11 cases should have been recorded in the target population. Patients discharged with AFP-compatible diagnoses identified from the hospital discharge records led to 30 cases of hospitalization with an incidence estimate of 2.72/100,000 (95% CI = 1.90–3.88/100,000). The estimated completeness of ascertainment by the ongoing surveillance system was therefore 14.15% (95% CI = 6.65–27.6, Table 1).

The average annual incidence of AFP, based on the capture-recapture method (Table 1), was estimated at 3.84 per 100,000 children under 15 years (95% CI = 2.84–5.19/100,000), with 3.69 cases/100,000 males <15 years old (95% CI = 3.47–7.35/100,000), and an annual incidence rate of 5.05/100,000 in females (95% CI = 2.41–5.64/100,000). A significant difference (*p* < 0.05) was calculated in the estimated probability of case ascertainment in females; in fact, only 3.7% (*N* = 1) of estimated cases have been ascertained in the surveillance system versus 23.8% (*N* = 5) of cases in males.

Considering all cases with a hospital admission, 70% (*N* = 21) had the 357.0 ICD-9-CM diagnosis code, which is used for “acute infective polyneuritis”, such as Guillain-Barré syndrome, 16.7% of patients (*N* = 5) had the 344.89 code, that is applied for “other specified paralytic syndrome” (es. flaccid paralysis), while 13% of them (*N* = 4) had the 341.22 code for “acute (transverse) myelitis NOS”.

## 4. Discussion

The results of the study show that routine surveillance by phone achieved an estimated 14.15% completeness of ascertainment, with an incidence estimate of 0.54 per 100,000, that does not reach the WHO target of 1 per 100,000 cases in children aged under 15 years. An increase in AFP ascertainment was obtained using HDRs, achieving an estimated 85.85% completeness of ascertainment, equivalent to an incidence estimate of 2.72/100,000 children under 15 years. These results are clearly less satisfactory than those recently reported from Egypt [20].

The two-source capture-recapture method led to an estimated average annual incidence of AFP of 3.84 per 100,000 children under 15 years of age. This value exceeds the WHO target and the incidence estimated by a previous study in the same region performed by using surveillance data alone but is in line with recent national estimates including the evaluation of HDR [8,21].

The capture-recapture method allows the estimation of the total size of a population by crossing different sources; it represents a helpful tool for estimating frequency, with an easy application and simple principles of calculation. However, there are conditions which must be satisfied to obtain accuracy and appropriateness of results. For example, one condition is that all the cases identified by each source are real. All the cases in the regional database of AFP are real and notified according to the WHO case definition established. In some cases, the identification of cases by HDRs can be difficult and challenging for the lack of a well-established specific definition [21]; in addition, procedures for coding data may differ between wards, potentially leading to an error in estimates [22]. However, the comparison of the two sources may be important to reduce this potential error. The sensitivity of the system may be even further improved through a “zero reporting system” or an e-mail reminder) to implement a more sensitive surveillance system, which also includes an environmental monitoring of the waste water, as reiterated in numerous and recent scientific evidences [23,24]. To note, the reasons for the estimated higher incidence of reporting AFP cases among males are unknown, but this finding might be related to either reporting biases or the presence of underlying factors, and has been recently registered in other international contexts [25,26,27,28]. This result is even more important as the estimated incidence rates among females and males do not differ significantly. However, besides using capture-recapture designs which have been frequently applied to assess the total case population of health conditions using different sources, we must underline the possible limitation of the above finding. In fact, to be valid, the capture-recapture methods need some assumptions: the study of a closed population; ability to perfectly match cases captured in both methods (in both methods, perfect classification); within a method, any has case equal probability of being captured; and independence of capture between the different surveillance systems [18].

The Lincoln–Petersen estimator may be biased at small sample sizes, like that used in this study. An alternative for small numbers is given by the Chapman estimator. One of the main limitations of this method is the assumption of independence between the two sources. In fact, it is known that with increasing dependencies between sources the Lincoln–Petersen/Chapman estimator will underestimate the population size; on the other hand, decreased dependencies will potentially overestimate a population size. Chao proposed an alternative estimator of population size which relaxes the assumption that identifying sources are independent; moreover Chao’s estimator has been shown to be more viable than Chapman’s estimator, and therefore should be carefully considered, especially in the context of dependency between sources [29,30,31]. In the present study we can assume that the two sources were quite independent; in fact, the routine notification system relied on paediatricians, regardless of the practice setting, while the HDR data were largely the responsibility of hospital medical or nursing staff. Moreover, all cases had an equal, a priori, probability of inclusion, given the absence of any theoretical barrier to the accessibility of hospital and community healthcare services in Italy. We think that cases had accurately been identified and linked and that appropriate matching occurred between sources. In fact, given the relatively limited number of observations, authors have been able to check the linkage between the two sources on a one-by-one basis; ambiguous results have been discussed and reviewed. However, we should consider that bias would have been reinforced if any of these assumptions were invalid.

## 5. Conclusions

During the reporting period, the notification system has not always reached the recommended notification target rate. Indeed, in the last 3 years examined (from 2012 to 2014), no case of AFP was notified by the phone surveillance. These results highlight the lack of exhaustiveness of the notification system in identifying AFP in children under 15 years and highlight the need for a change in the process.

Vaccine-preventable diseases continue to pose a great risk to the community and the hospital setting. [29,30] Knowledge of signs and symptoms, awareness of outbreaks in-community and elsewhere, and early consultation with an expert in infectious diseases and public health authorities in suspected cases, are key preventive care measures [32,33,34,35].

Moreover, enhancing the surveillance system will be a key to achieve a prompt identification of viruses potentially causing serious disease in the community.

Surveillance has been supported by the Italian National Institute of Health.

## Figures and Tables

**Table 1:** Crude and stratified analysis by year of acute flaccid paralysis (AFP) ascertainment: cases ascertained (notified and in hospital discharge records HDRs) and estimated total cases.

	Notified Cases (Primary Source) (a)	AFP Cases in HDRs (Secondary Source) (b)	Total Observed Cases by Both Sources (c)	Estimated Total Number of Cases (*N*)	Estimated Completeness of AFP Surveillance System (d = a/*N* × 100)	Estimated Incidence from the Capture-Recapture Method per 100,000 Children under 15
Year						
2010	0	4	0	4	0.00	1.81 (95% CI = 0.70–4.66)
2011	5	9	3	14	35.71	6.3 (95% CI = 3.75–10.58)
2012	1	6	1	6	16.67	1.81 (95% CI = 0.70–4.66)
2013	0	7	0	7	0.00	3.18 (95% CI = 1.55–6.56)
2014	0	4	0	4	0.00	1.82 (95% CI = 0.71–4.67)
Age group						
0–3 years	3	14	2	19	15.79	6.83 (95% CI = 4.37–10.67)
4–7 years	3	5	2	7	42.86	2.52 (95% CI = 1.22–5.19)
8–11 years	0	3	0	3	0.00	1.10 (95% CI = 0.37–3.22)
12–15 years	0	8	0	8	0.00	2.92 (95% CI = 1.48–5.76)
Sex						
Females	1	13	0	27	3.7	5.05 (95% CI = 3.47–7.35)
Males	5	17	4	21	23.8	3.69 (95% CI = 2.41–5.64)
Total	6	30	4	37	16.2	4.40 (95% CI = 3.18–5.77)

## Appendix A

**Table A1:** List of diagnostic ICD-9 codes compatible with diagnosis of AFP.

ICD-9-CM Diagnosis Code	Diagnosis
357.0	Acute infective polyneuritis (includes Guillain-Barré syndrome)
045.0	Acute paralytic poliomyelitis specified as bulbar
045.00	Acute paralytic poliomyelitis specified as bulbar, poliovirus, unspecified type
045.01	Acute paralytic poliomyelitis specified as bulbar, poliovirus type I
045.02	Acute paralytic poliomyelitis specified as bulbar, poliovirus type II
045.03	Acute paralytic poliomyelitis specified as bulbar, poliovirus type III
045.1	Acute poliomyelitis with other paralysis
045.10	Acute poliomyelitis with other paralysis, poliovirus, unspecified type
045.11	Acute poliomyelitis with other paralysis, poliovirus type I
045.12	Acute poliomyelitis with other paralysis, poliovirus type II
045.13	Acute poliomyelitis with other paralysis, poliovirus type III
045.2	Acute nonparalytic poliomyelitis
045.20	Acute nonparalytic poliomyelitis, poliovirus, unspecified type
045.21	Acute nonparalytic poliomyelitis, poliovirus type I
045.22	Acute nonparalytic poliomyelitis, poliovirus type II
045.23	Acute nonparalytic poliomyelitis, poliovirus type III
045.9	Unspecified acute poliomyelitis
045.90	Acute poliomyelitis, unspecified, poliovirus, unspecified type
045.91	Acute poliomyelitis, unspecified, poliovirus type I
045.92	Acute poliomyelitis, unspecified, poliovirus type II
045.93	Acute poliomyelitis, unspecified, poliovirus type III
341.2	Acute (transverse) myelitis
341.20	Acute (transverse) myelitis Not Otherwise Specified (NOS)
341.21	Acute (transverse) myelitis in conditions classified elsewhere
341.22	Idiopathic transverse myelitis
344.89	Other specified paralytic syndrome (includes flaccid paralysis)
